# The Medical Annual
*"The Medical Annual for 1890." (Bristol: John Wright and Co.; London: Simpkin, Marshall, Hamilton, Kent, and Co.)


**Published:** 1890-04-05

**Authors:** 


### THE PRACTITIONER'S BOOKSHELF.

(Books for Review should be sent to The Editor, The Lodge, Porchester Square, W.)

### THE MEDICAL ANNUAL.*

This very useful compendium has reached its eighth year of publication. In addition, we find from the preface that there is an American edition. Our experimental knowledge increases so fast that the editors have been compelled to enlarge their volume to 656 pages. It still remains, however, a very handy book, and the increase of firmness in the binding makes it much more comfortable in its use. The following list of contributors is sufficient evidence of the value of the work: H. W. Allingham, F.R.C.S. (Surgery of Rectum); R. S. Fancourt Barnes, M.D. (Perinœorrhaphy); H. C. Chakin, M.A., M.D. (Diseases of Children); J. Michell Clarke, M.A., M.D. (Locomotor Ataxy); Surgeon-Major Crombie, M.D. (Diseases of India); Charles L. Dana, M.D. (Diseases of Nervous System); Dr. S. Davies, M.B., D.P.H. (Sanitation); W. H. Elam, F.R.C.S. (Cancer and Cerebral Surgery); E. Hurry Fenwick, F.R.C.S. (Diseases of Bladder, Prostate, and Urethra); J. Dundas Grant, M.A., M.D. (Diseases of Ear); George Harley, M.D. (Gall Stones); F. De Havilland Hall, M.D. (Fevers and General Medicine); F. W. Hewitt, M.A., M.D. (Anæsthesia); Jonathan Hutchinson, F.R.C.S. (Venereal Diseases); Frank W. Jackson, M.D. (Diseases of the Heart); W. Allan Jamieson, M.D. (Diseases of the Skin); Skene Keith, M.B. (Uterine Electro-therapeutics); W. Lang, F.R.C.S. (Diseases of the Eye); James R. Leaming, M.D. (Diseases of the Heart); A. H. N. Lewens, M.D. (Obstetrics); Greville MacDonald, M.D. (Diseases of the Nose); Sidney Martin, M.D. (Dictionary of New Remedies); Walter Pye, F.R.C.S. (General Surgery); A. W. Mayo Robson, F.R.C.S. (Abdominal Surgery); A. D. Rockwell, M.D. (Electro-Therapeutics); Bernard Rott, F.R.C.S. (Orthopædics); Robert Saundby, M.D. (Diseases of the Kidney); R. Shingleton Smith, M.D. (Phthisis); J. W. Taylor, F.R.C.S. (Diseases of Women); C. Lloyd Tuckey, M.D. (Hypnotism); Wm. Walter, M.A., M.D. (Cancer of the Uterus); Lionel A. Weatherly, M.D. (Insanity); F. J. Wethered, M.D. (Diseases of the Lungs); Percy Wilde, M.D. (Thermo-Therapeutics); *P. Watson Williams, M.B. (Diseases of the Throat); Graham Wills, M.D. (Therapeutic Synopsis). There is a very full and complete "Dictionary of New Remedies and Review of Therapeutic Progress for the Year 1889," by Dr. Sidney Martin; a long chapter on the virtues of hot and cold water in the various forms of bathing, under the heading of "Thermo-Therapeutics," by Dr. Percy Wilde; and a readable chapter on "Electro-Therapeutics," by Dr. Rockwell, of New York. The main body of the work is made up of descriptions of new treatment. This occupies over 400 pages, and is by the many experienced hands whose names are given above. Part III. comprehends "Sanitary Science," by Mr. O. S. Davies, M.B., the Medical Officer of Health for Bristol, who gives us a very practical compilation. Then follows a list of lunatic asylums and homes for inebriates in Great Britain and Ireland. There are chapters on new inventions and progress of pharmacy, a list of the chief medical works published in the year, a short account of life assurance, a useful official and trade directory, with a few blank pages at the end for notes, and there are a good many useful illustrations and diagrams scattered throughout the book. We are glad to notice a distinct advance in the value of this publication, and venture to utter but one slight protest against the insertion of advertisements in the pages of the subject matter of the book—a practice perhaps commercially advantageous, but vexatious to the ordinary reader.

* "The Medical Annual for 1890." (Bristol: John Wright and Co.; London: Simpkin, Marshall, Hamilton, Kent, and Co.)

* Secretary also to the Editorial Committee.

